# Activation of the JNK/COX-2/HIF-1α axis promotes M1 macrophage via glycolytic shift in HIV-1 infection

**DOI:** 10.26508/lsa.202302148

**Published:** 2023-10-05

**Authors:** Junhan Zhang, Zongxiang Yuan, Xuanrong Li, Fengyi Wang, Xueqin Wei, Yiwen Kang, Chuye Mo, Junjun Jiang, Hao Liang, Li Ye

**Affiliations:** Guangxi Key Laboratory of AIDS Prevention and Treatment, School of Public Health, Guangxi Medical University , Nanning, China

## Abstract

Chronic inflammation is recognized as a major risk factor for the severity of HIV infection. Whether metabolism reprogramming of macrophages caused by HIV-1 is related to chronic inflammatory activation, especially M1 polarization of macrophages, is inconclusive. Here, we show that HIV-1 infection induces M1 polarization and enhanced glycolysis in macrophages. Blockade of glycolysis inhibits M1 polarization of macrophages, indicating that HIV-1–induced M1 polarization is supported by enhanced glycolysis. Moreover, we find that this immunometabolic adaptation is dependent on hypoxia-inducible factor 1α (HIF-1α), a strong inducer of glycolysis. HIF-1α–target genes, including HK2, PDK1, and LDHA, are also involved in this process. Further research discovers that COX-2 regulates HIF-1α–dependent glycolysis. However, the elevated expression of COX-2, enhanced glycolysis, and M1 polarization of macrophages could be reversed by inactivation of JNK in the context of HIV-1 infection. Our study mechanistically elucidates that the JNK/COX-2/HIF-1α axis is activated to strengthen glycolysis, thereby promoting M1 polarization in macrophages in HIV-1 infection, providing a new idea for resolving chronic inflammation in clinical AIDS patients.

## Abstract (precis)

This study reveals JNK/COX-2/HIF-1α axis activation enhances glycolysis, promoting M1 polarization in HIV-1–infected macrophages, offering a novel approach to AIDS-related inflammation.

## Graphical Abstract

**Figure:**
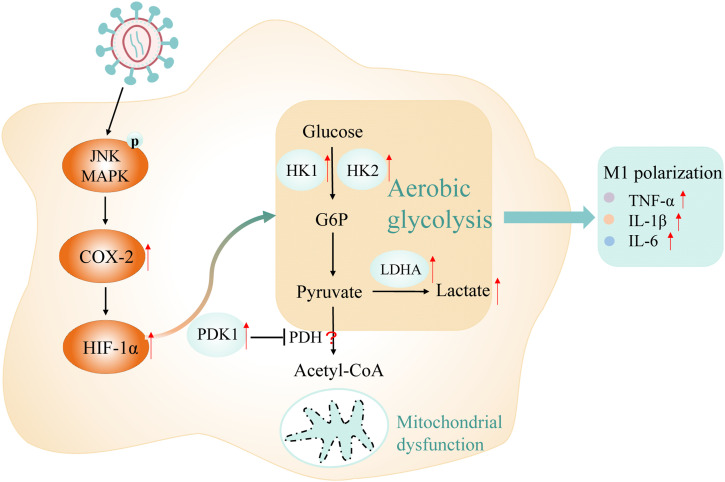

## Introduction

Chronic inflammation is a hallmark of HIV-1 infection, and it persists even when combination antiretroviral therapy is effective (Deeks et al, 2013). This persistent inflammation is now widely recognized as a major risk factor for the severity of HIV infection, which can exacerbate tissue damage and lead to HIV-related non-communicable complications such as cardiovascular diseases, liver and kidney diseases, malignant tumors, and potentially fatal outcomes (Deeks et al, 2013). Although some anti-inflammatory drugs have been applied clinically to address chronic inflammation in HIV-1–infected patients, the therapeutic effect is not satisfactory (O’Brien et al, 2017; Utay & Overton, 2020). Therefore, there is an urgent need to better understand the mechanisms driving chronic inflammation in HIV-1 infection and to discover new therapeutic intervention targets.

In the past few years, the role of cellular metabolism in the fate and activity of immune cells has been revealed, including its influence on the outcome of infectious diseases. Increasing evidence indicates that immunometabolism plays an important role in HIV-1 pathogenesis. It is believed that HIV-1 infection is favored in cells with high levels of metabolic activities (Sáez-Cirión et al, 2021). Some studies have shown that HIV-1–infected CD4^+^ T cells are characterized by increased glycolysis and glutaminolysis, which support HIV-1 infection (Hegedus et al, 2014, 2017). On the contrary, different metabolic characteristics of HIV-specific CD8^+^ T cells from non-controllers and from HIV controllers (HICs) have been revealed. Based on this, researchers proposed to reprogram the metabolic profile of CD8^+^ T cells as a strategy for HIV-1 cure (Angin et al, 2019; Perdomo-Celis et al, 2022). As in T cells, altered metabolic profiles have also been observed in HIV-1–infected macrophages (Datta et al, 2016). Chronic inflammation in HIV-1 infection is closely related to the activity of immune cells, particularly macrophages.

Macrophages are not only the target cells of HIV-1 infection, but also the key regulators of chronic immune activation in HIV/AIDS patients, especially in the advanced stage of HIV-1 infection. Because of their longevity, macrophages play a more prominent immunomodulatory role compared with the depleted T cells (Cashin et al, 2011). In response to microenvironmental changes, macrophages undergo polarization. This polarization process, resulting in classically activated (M1) and alternatively activated (M2) macrophages, is critical in mediating effective immune responses against pathogen invasion. M1 macrophages participate in pro-inflammatory response after being activated by Th1 cytokines, pro-inflammatory cytokines, and chemokines, whereas M2 macrophages promote anti-inflammatory response and tissue damage repair (Cassol et al, 2009). Some pathogens can use these activation pathways to facilitate dissemination and pathogenesis. As for HIV-1, imbalanced polarization of macrophages is a key mechanism for chronic inflammation and immune activation in HIV/AIDS patients (Teer et al, 2021).

Along with polarization, macrophages initiate adaptive reprogramming of metabolism in response to antigen stimuli through rapid changes in key genes and enzymes in metabolic pathways (O’Neill et al, 2016; Saha et al, 2017). Some studies have demonstrated that macrophage polarization is tightly bound up with the alteration in glucose metabolism. For example, LPS-induced M1 macrophages use glycolysis as the main energy supplier because of the high demand for rapid ATP synthesis. This phenomenon is similar to the well-known Warburg effect or aerobic glycolysis (Palsson-McDermott et al, 2015). In contrast, in M2 macrophages, oxidative phosphorylation (OXPHOS) can better meet the continuous intracellular energy supply required by macrophages for tissue repair and wound healing (Viola et al, 2019; Wang et al, 2018). An earlier study showed that HIV infection can induce unique metabolic characteristics in macrophages, including lipid accumulation and reduced mitochondrial ATP production (Castellano et al, 2019). In addition, metabolic reprogramming, as a result of HIV-1 infection, is conducive to the regulation of the cellular immune function (Sáez-Cirión et al, 2021). As for macrophages, the inherent plasticity provides a basis for macrophage-centered therapeutic approaches (Kim et al, 2021). However, there is limited information on the precise mechanism(s). Therefore, it is very attractive to obtain in-depth insights into the effects of metabolic reprogramming on macrophage polarization in HIV-1 infection.

In this study, we investigated the polarization state of macrophages upon HIV-1 infection and found that the M1 subtype was induced along with the secretion of various pro-inflammatory cytokines. In the metabolic analysis, we found that glucose metabolic reprogramming occurred in HIV-1 infection, with enhanced glycolysis and reduced TCA and OXPHOS. Mechanistically, we found that HIV-1 activates JNK/COX-2/hypoxia-inducible factor 1α (HIF-1α) signaling to alter glucose metabolism, thereby facilitating M1 polarization in macrophages. Our results reveal new understandings of immunometabolism in macrophages in response to HIV-1 infection and provide novel strategies for clinical intervention of chronic inflammation in HIV/AIDS patients.

## Results

### HIV-1 infection induces M1 polarization of macrophages

**Figure 1.**
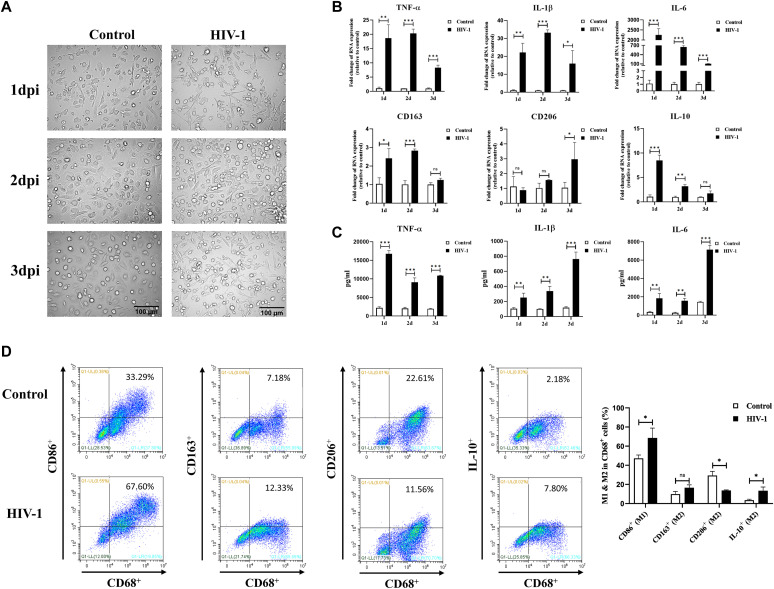
HIV-1 infection induces M1 polarization of macrophages. **(A)** Morphologic features of monocyte-derived macrophages (MDMs) after HIV-1 infection for 1–3 d. Scale bar, 100 μm. **(B, C)** Changes in mRNA (B) and protein expressions (C) of M1 and M2 polarization markers in MDMs after HIV-1 infection for 1–3 d. (Statistical analysis was performed using a *t* test, *P < 0.05, **P < 0.01, and ***P < 0.001.) **(D)** Representative results of expressions of M1 and M2 polarization markers in MDMs detected by flow cytometry. (Statistical analysis was performed using a *t* test, *P < 0.05).

**Figure S1.**
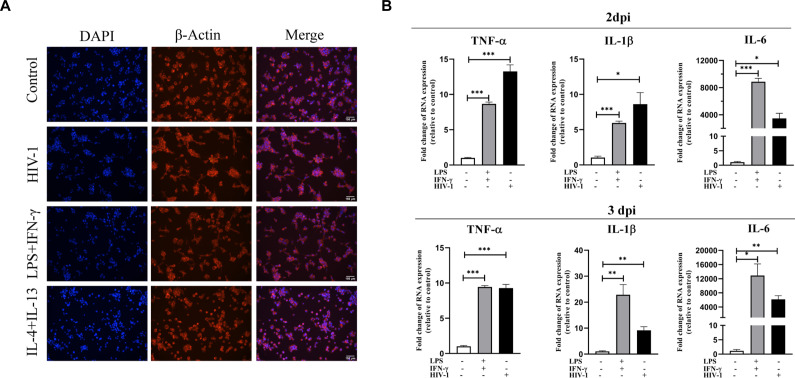
Impacts of HIV-1 on monocyte-derived macrophages are similar to LPS and IFN-γ co-stimulation. THP-1 cells were seeded in a culture plate with PMA (50 μM) stimulation (48 h) to differentiate macrophages. LPS (1 μg/ml) and IFN-γ (20 mg/ml) were co-used for typical M1 polarization, whereas IL-4 (20 mg/ml) was used in combination with IL-10 (20 mg/ml) for M2 polarization. **(A)** Immunofluorescence staining of DAPI (representing nucleus) and β-actin (representing cytoskeleton) was used to observe morphological changes in macrophages with different stimuli for 48 h. Scale bar, 150 μm. **(B)** RT–qPCR analysis showed the mRNA levels of TNF-α, IL-1β, and IL-6 in THP-1 macrophages after 2 d (upper) and 3 d (bottom) of stimulation. (Statistical analysis was performed using a *t* test, *P < 0.05, **P < 0.01, and ***P < 0.001).

THP-1 cells or monocytes from healthy donors were respectively differentiated into macrophages in vitro, for the following infection experiments. The morphology of HIV-1–infected monocyte-derived macrophages (MDMs) under an inverted optical microscope showed that macrophages gradually developed an irregular shape and had prolonged long pseudopodia post–HIV-1 infection (Fig 1A), similar to LPS-stimulated M1 macrophages (Fig S1A). In addition, the mRNA expression of M1 markers, including TNF-α, IL-1β, and IL-6, was significantly elevated in HIV-1–infected cells compared with the control group (Figs 1B and S1B). Although the M2 markers CD163, CD206, and IL-10 increased simultaneously, the magnitude of the increase was much lower than that of the M1 markers (Fig 1B). At the protein level, we observed these M1 inflammatory factors (TNF-α, IL-1β, and IL-6) sustained the release to the supernatant during HIV-1 infection (Fig 1C). Furthermore, flow cytometric analyses revealed an increased proportion of CD68^+^CD86^+^ M1 macrophages after HIV-1 infection (33.29% versus 67.60%), whereas the proportion of CD68^+^CD163^+^ M2 macrophages was just slightly increased (7.18% versus 12.33%), and the proportion of CD68^+^CD163^+^ and CD68^+^IL-10^+^ M2 macrophages was decreased after HIV-1 infection (22.61% versus 11.56%; 2.18% versus 7.80%, respectively) (Fig 1D). Taken together, the above evidence confirms that HIV-1 infection induces M1 polarization of macrophages.

### Changes in glucose metabolism in HIV-1–infected macrophages

**Figure 2.**
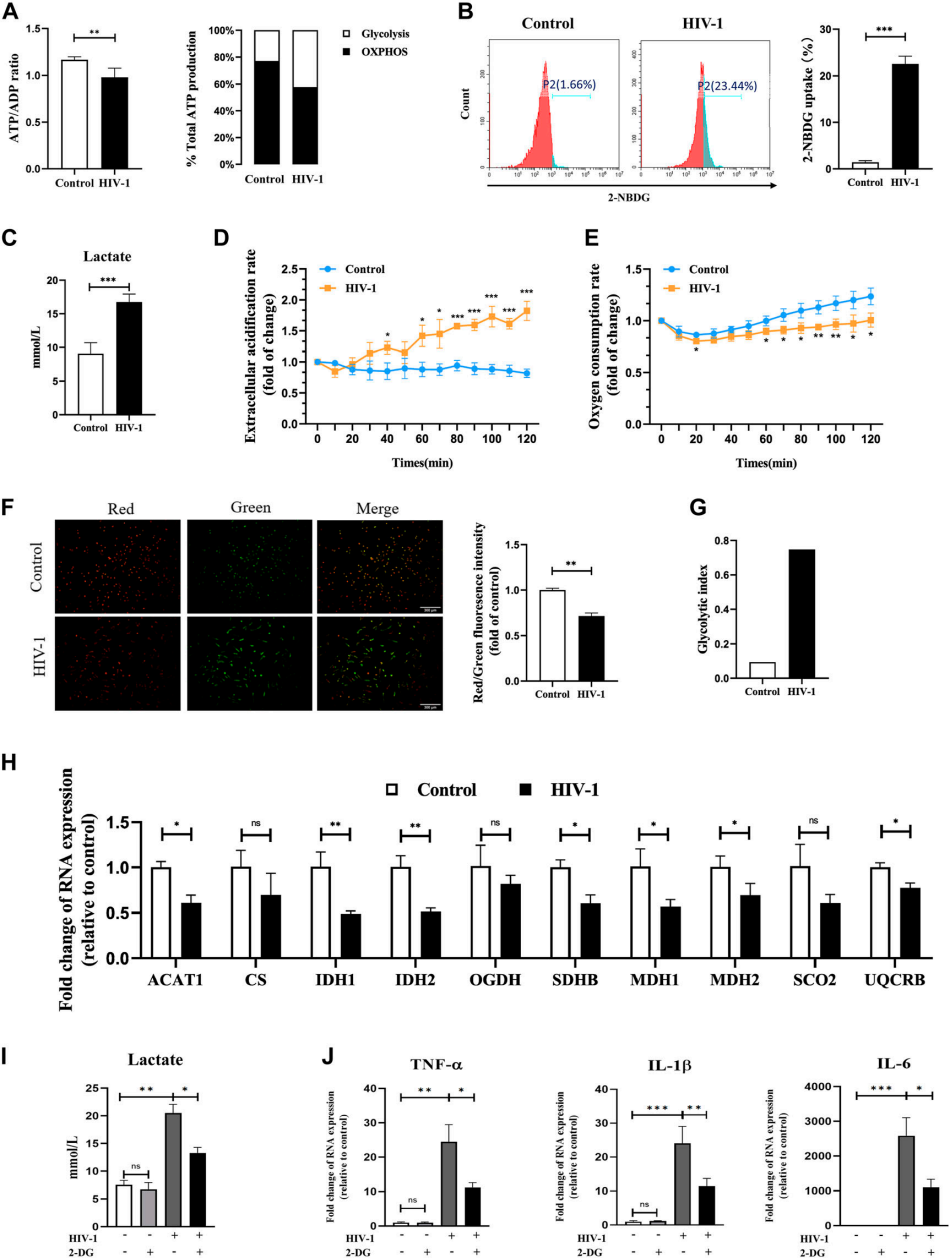
Metabolism reprogramming affects polarization in HIV-1–infected macrophages. **(A)** ATP/ADP ratio (left panel) and relative ATP production from glycolysis and oxidative phosphorylation (right panel) in monocyte-derived macrophages (MDMs) infected or uninfected with HIV-1. (Statistical analysis was performed using a *t* test, *P < 0.05, **P < 0.01, and ***P < 0.001.) **(B)** Representative results of glucose uptake analyzed by flow cytometry. (Statistical analysis was performed using a *t* test, ***P < 0.001.) **(C)** Lactate concentration in the supernatant of MDMs infected with HIV-1 for 48 h was detected using colorimetry. (Statistical analysis was performed using a *t* test, ***P < 0.001.) **(D, E)** Extracellular acidification rate and oxygen consumption rate of MDMs infected with HIV-1 for 48 h. Time-resolved fluorescence was applied and monitored for 120 min. Results were relative to 0 min. (Statistical analysis was performed using a *t* test, *P < 0.05, **P < 0.01, and ***P < 0.001.) **(F)** Mitochondrial membrane potential was assessed using the JC-1 assay. A change from red to green fluorescence indicates a decrease in the mitochondrial membrane potential. The red/green fluorescence ratio was calculated for comparison. (Statistical analysis was performed using a *t* test, **P < 0.01.) Scale bar, 300 μm. **(G)** Bar plot showed glycolytic index (GI) differences in control and HIV-1–infected MDMs. **(H)** mRNA expressions of key genes in TCA and oxidative phosphorylation. (Statistical analysis was performed using a *t* test, ns non-significant, *P < 0.05, **P < 0.01, and ***P < 0.001.) **(I, J)** MDMs were pretreated with or without 2-DG (10 mM) for 2 h, followed by infection of HIV-1 for 48 h. **(I, J)** Lactate concentration in the culture supernatant (I), and mRNA expressions of TNF-α, IL-1β, and IL-6 (J) were detected. (Statistical analysis was performed using a *t* test, *P < 0.05, **P < 0.01, and ***P < 0.001).

Previous studies have shown that the metabolic reprogramming is involved in polarization of macrophages (Russell et al, 2019). Changes in the ATP/ADP ratio can indicate the primary energy metabolic pathway in the cell (Maldonado & Lemasters, 2014; Tan et al, 2022). Therefore, we used the ATP/ADP ratio to determine the metabolic state of HIV-1–infected macrophages. As a result, a lower ATP/ADP ratio was observed under HIV-1 infection, which indicated the cell metabolism is predominantly glycolytic (Fig 2A, left panel). Besides that, we measured the relative ATP production from glycolysis and OXPHOS in the context of HIV-1 infection in macrophages via inhibition of two ATP production pathways. Inhibitor 2-DG (10 mM), targeting hexokinase II (HK2), was used to block glycolysis (Pajak et al, 2019), and inhibitor oligomycin (40 μM) was used to block mitochondrial ATP production (Nieminen et al, 1994). Inhibition of glycolysis with 2-DG resulted in a greater ATP reduction in HIV-1–infected MDMs compared with control cells (HIV-1: 51.2% versus Control: 40.6%), whereas oligomycin inhibition of mitochondrial ATP production had a greater effect on reduction of ATP in uninfected control cells (Fig 2A, right panel), revealing that HIV-1 facilitates the use of glycolysis for energy supply.

To verify the changes in glucose metabolism pathways by HIV-1 infection, glucose uptake was detected. The results showed a significant increase in the proportion of glucose uptake in HIV-1–infected cells compared with control cells (HIV-1: 23.44% versus Control: 1.66%) (Fig 2B). As the final metabolic product of glycolysis, lactate accumulation means the increase in glycolysis. In the HIV-1–infected group, the level of lactate in the supernatant was about 1.7 times that in the control group (Fig 2C), and the results of the extracellular acidification assay yielded identical results (Fig 2D). More significantly, the latter showed a time-dependent effect. Meanwhile, we performed the oxygen consumption assay and the JC-1 mitochondrial membrane potential (MMP) assay to estimate the mitochondrial activity during HIV-1 infection. HIV infection led to a significant decrease in the oxygen consumption rate (OCR) and a 40% reduction in MMP, indicating impaired mitochondrial activity (Fig 2E and F). By calculating glycolytic indexes (lactate production × glucose uptake rate/OCR) (Valentín-Guillama et al, 2018), comprehensively, we revealed that glycolysis was enhanced in HIV-1–infected MDMs (Fig 2G).

In addition, we detected a few key genes for TCA and OXPHOS in HIV-1–infected MDMs. Intriguingly, the expression of all these genes was down-regulated. Among them, isocitrate dehydrogenase (IDH) was down-regulated by about two times (*P* < 0.01), and malate dehydrogenase was down-regulated by about 1.6 times (*P* < 0.05) (Fig 2H). IDH and malate dehydrogenase are both important rate-limiting enzymes in the TCA cycle. IDH catalyzes the synthesis of α-ketoglutarate, which is the intermediate product connecting carbon metabolism and nitrogen metabolism in the body.

The above results suggest that HIV-1 infection may alter the glucose metabolism of macrophages by promoting glycolysis and inhibiting TCA and OXPHOS.

### Suppression of glycolysis blocks M1 polarization of macrophages in HIV-1 infection

**Figure S2.**
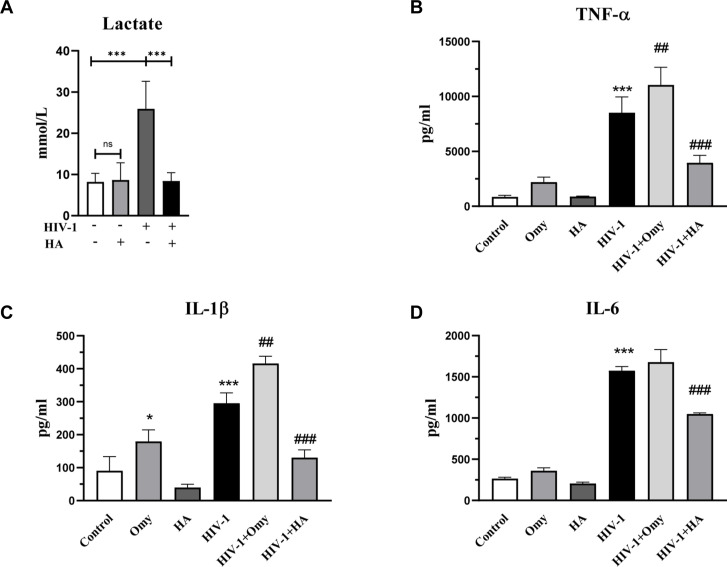
Heptelidic acid inhibits glycolysis and inflammation induced by HIV-1 infection, whereas oligomycin exerts the opposite effect. Monocyte-derived macrophages were treated with 10 μM heptelidic acid (HA) or 40 μM oligomycin (Omy) for 8 h during the 48 h of HIV-1 infection. **(A, B, C, D)** Lactate concentration in the culture supernatant (A), and expressions of TNF-α, IL-1β, and IL-6 (B, C, D) were detected. (Statistical analysis was performed using a *t* test, *P < 0.05, **P < 0.01, and ***P < 0.001 as compared to Control; #*P* < 0.05, ##*P* < 0.01, and ###*P* < 0.001 as compared to HIV-1).

In multiple reports, M1 macrophages are characterized by increased glycolytic activity, whereas M2 macrophages are characterized by increased fatty acid oxidation (FAO), glutaminolysis, and mitochondrial respiration (Viola et al, 2019). To investigate the impact of metabolic reprogramming in HIV-1–induced M1 polarization, we used 2-DG (targeting HK2) and heptelidic acid (targeting GAPDH) to block glycolysis. As shown in Figs 2I and S2A, both 2-DG and heptelidic acid treatment effectively reduced the elevated lactate release by HIV-1 infection. Under the condition of glycolysis inhibition, we observed that the expression of M1 markers TNF-α, IL-1β, and IL-6 was also reversed (Figs 2J and S2B–D), indicating that blocking glycolysis inhibits M1 polarization of macrophages driven by HIV-1 infection. In contrast, treatment of oligomycin promoted the release of pro-inflammatory cytokines, as evidenced in Fig S2.

### HIV-1 infection leads to immunometabolic reprogramming in macrophages via activation of HIF-1α

**Figure 3.**
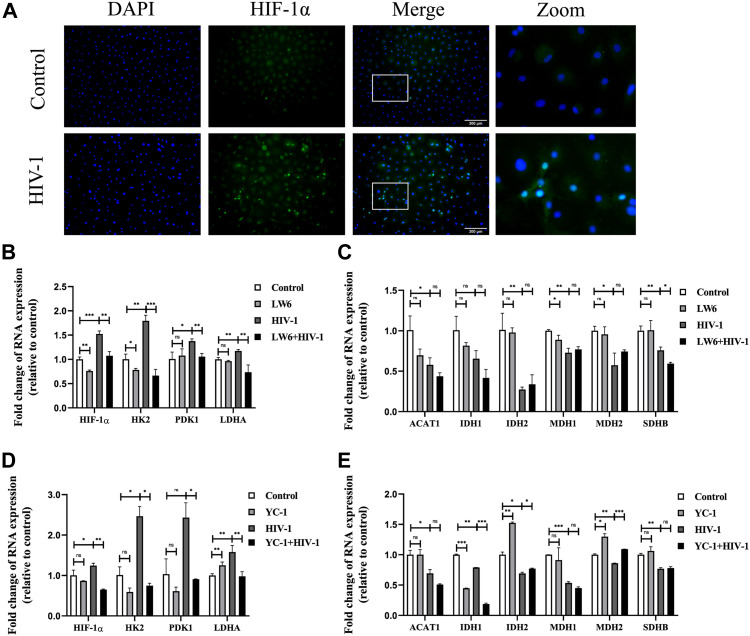
Activation of HIF-1ɑ impinges upon gene regulation of glucose metabolism. **(A)** Immunofluorescence showed HIF-1ɑ protein localization and expression in monocyte-derived macrophages (MDMs). Blue: DAPI; green: HIF-1ɑ. Scale bar, 300 μm. **(B, C)** MDMs were pretreated with or without LW6 (15 μM) for 2 h, followed by HIV-1 infection for another 48 h. **(B, C)** RT–qPCR analysis showed the mRNA levels of key genes in the glycolysis (B) and TCA cycle (C). (Statistical analysis was performed using a *t* test, *P < 0.05, **P < 0.01, and ***P < 0.001.) **(D, E)** MDMs treated with or without YC-1 (1 μM) and infected with or without HIV-1 were assessed for expressions of key genes in the glycolysis (D) and TCA cycle (E). (Statistical analysis was performed using a *t* test, *P < 0.05, **P < 0.01, and ***P < 0.001).

**Figure 4.**
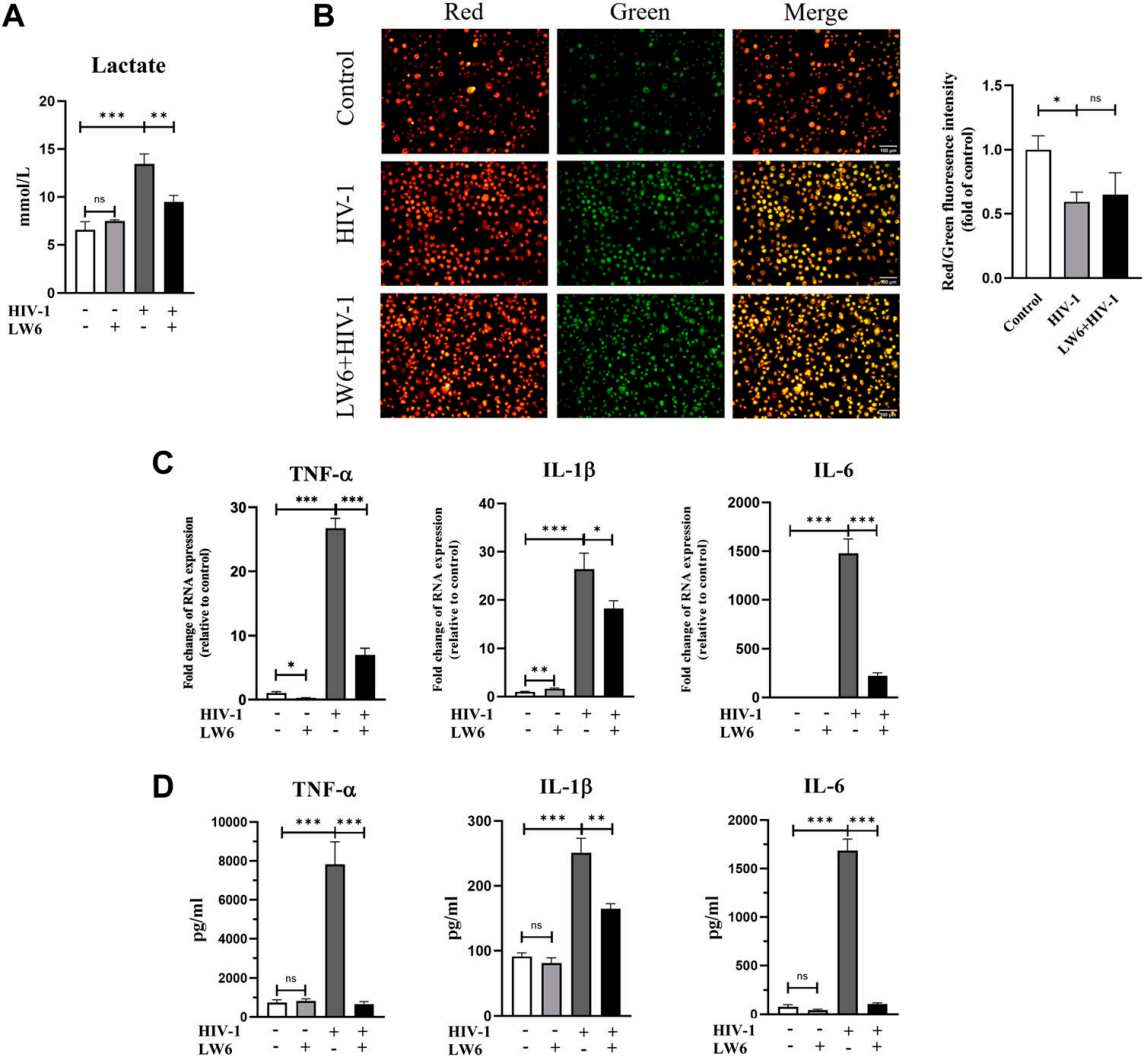
Effect of HIF-1ɑ inhibition on glycolysis, mitochondrial activity, and M1 polarization in monocyte-derived macrophages (MDMs). MDMs were infected with HIV-1 for 48 h, in the presence or absence of LW6 (15 μM) pretreatment (2 h). **(A)** Lactate concentration in the culture supernatant of MDMs. (Statistical analysis was performed using a *t* test, **P < 0.01 and ***P < 0.001.) **(B)** Mitochondrial membrane potential was assessed by the JC-1 assay. (Statistical analysis was performed using a *t* test, *P < 0.05.) Scale bar, 100 μm. **(C, D)** mRNA (C) and protein levels (D) of TNF-α, IL-1β, and IL-6 were detected using RT–qPCR and ELISA, respectively. (Statistical analysis was performed using a *t* test, *P < 0.05, **P < 0.01, and ***P < 0.001).

To further study the molecular mechanism of immunometabolic reprogramming by HIV-1 infection, we investigated the expression of HIF-1α, a known strong inducer of glycolysis, in HIV-1–infected MDMs. Immunofluorescence results showed that HIF-1α expression was enhanced after HIV-1 infection and was mainly located in the nucleus (Fig 3A). HIF-1α–activating genes, including HK2, PDK1, and LDHA (which function vitally in the glycolytic pathway), were also up-regulated by HIV-1 infection. However, in the presence of LW6 (15 μM), an inhibitor of HIF-1α, the HIV-1–induced up-regulation of HIF-1α, as well as its downstream targets HK2, PDK1, and LDHA, decreased to levels comparable to those in the control group (Fig 3B). The changes in expression of these factors at the protein level were also revalidated by Western blot, as shown in Fig 8B. As for key genes of OXPHOS, LW6 treatment had little effect on the down-regulated expression of TCA key genes induced by HIV-1 infection (Fig 3C). Meanwhile, treatment of another HIF-1α inhibitor YC-1 (1 μM) obtained similar results (Fig 3D and E). Next, we performed a lactate test and the JC-1 assay to determine the role of HIF-1α in HIV-1–induced glucose metabolic reprogramming. As shown in Fig 4A and B, elevated lactate induced by HIV-1 infection was effectively restrained under LW6 treatment, whereas the impaired mitochondrial activity was not restored, suggesting that HIF-1α activation contributes to glycolysis enhancement, but may not be a direct factor for the OXPHOS reduction in HIV-1 infection. In addition, we observed decreased expressions of TNF-α, IL-1β, and IL-6 after HIF-1α inhibition, both at mRNA (Fig 4C) and protein (Fig 4D) levels. Collectively, the above evidence suggests that HIV-1 infection activates the HIF-1α pathway to promote glycolysis and cause immunometabolic reprogramming in macrophages.

### HIV-1 regulates HIF-1α–dependent immunometabolic reprogramming via COX-2

**Figure 5.**
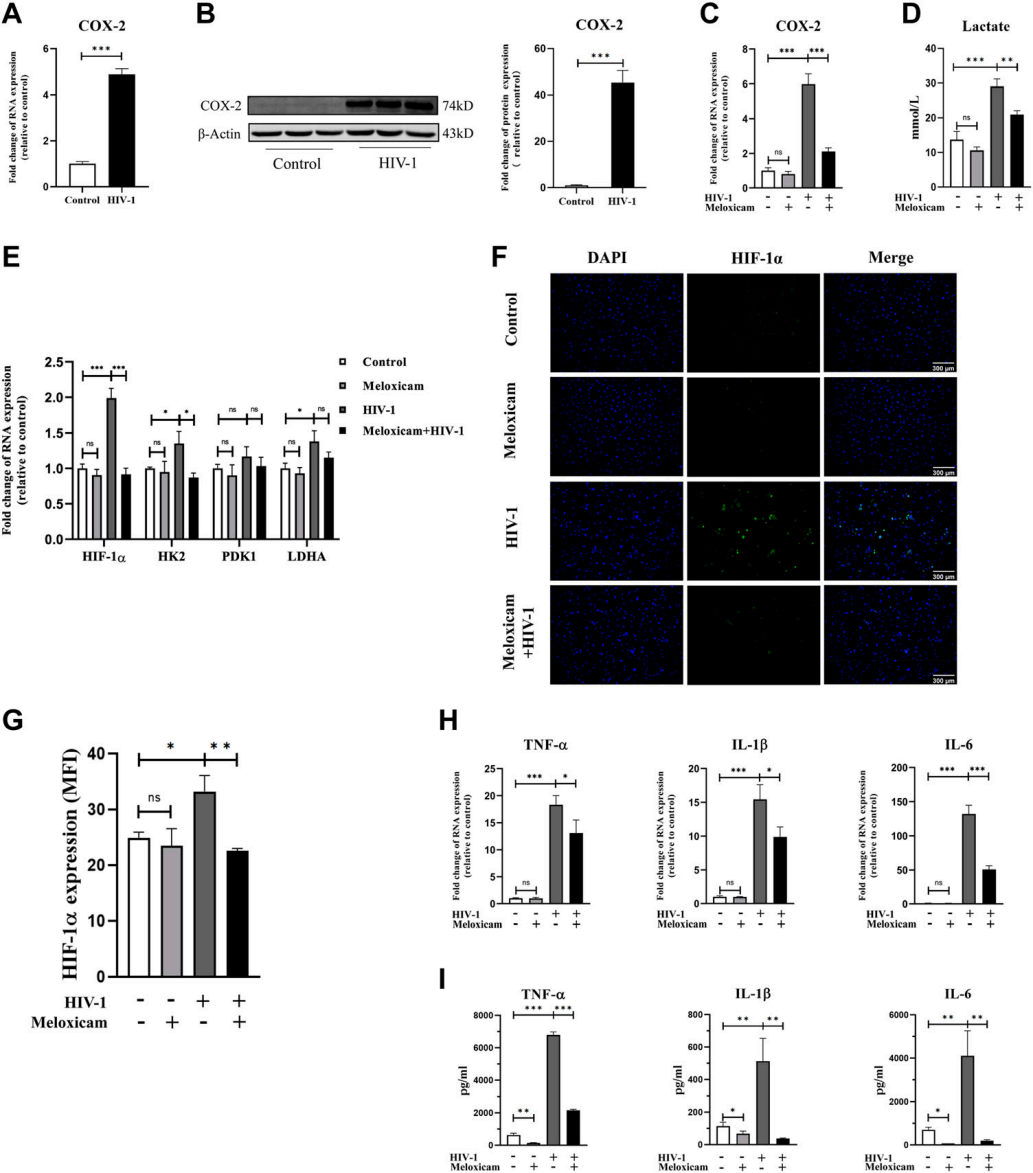
Activation of COX-2 mediates HIF-1ɑ–dependent glucose metabolism reprogramming and M1 polarization in monocyte-derived macrophages (MDMs). **(A, B)** mRNA (A) and protein expressions (B) of COX-2 in MDMs after HIV-1 infection for 48 h. β-Actin was used as the normalization control in Western blot analysis. (Statistical analysis was performed using a *t* test, **P < 0.001.) **(C, D, E, F, G, H, I)** MDMs were pretreated with meloxicam (50 μM) for 2 h; then, HIV-1 particles were added to incubate for another 48 h. COX-2 expression (C), lactate production (D), key glycolytic gene expression (E), hypoxia-inducible factor 1α expression ((F), scale bar, 300 μm), and M1 polarization (H, I) were measured. **(G)** Quantification of hypoxia-inducible factor 1α fluorescence intensity. (Statistical analysis was performed using a *t* test, *P < 0.05, **P < 0.01, and ***P < 0.001).

Previous studies have demonstrated COX-2 activation motivates pro-inflammation, which is the main manifestation of M1 macrophages (Simon, 1999). In HIV-1–infected macrophages, we found COX-2 mRNA and protein expressions were markedly up-regulated (Fig 5A and B). Therefore, we sought to investigate whether the up-regulation of COX-2 is involved in mediating HIV-1–induced immunometabolic reprogramming in macrophages. Using meloxicam (50 μM), a selective inhibitor of COX-2 synthesis (Engelhardt, 1996), we found that meloxicam significantly reduced COX-2 expression induced by HIV-1 infection (Fig 5C). Key markers of glycolysis were subsequently detected. Results revealed that meloxicam treatment reversed the HIV-1–induced lactate production (Fig 5D), and also reversed the increased expression of HIF-1α and key enzymes in glycolysis (HK2, PDK1, and LDHA), resulting in a rate of glycolysis comparable to uninfected MDMs (Figs 5E and 8B). In addition, results of immunofluorescence exhibited obviously weakened fluorescence of HIF-1α after meloxicam treatment (Fig 5F and G), suggesting that COX-2 regulates the enhancement of glycolysis during HIV-1 infection through the HIF-1α pathway.

We subsequently correlated COX-2 activation with M1 polarization, showing that the expression of HIV-1–induced pro-inflammatory cytokines (TNF-α, IL-1β, and IL-6) was notably reversed by meloxicam treatment, particularly at the level of protein expression (Fig 5H and I).

**Figure 6.**
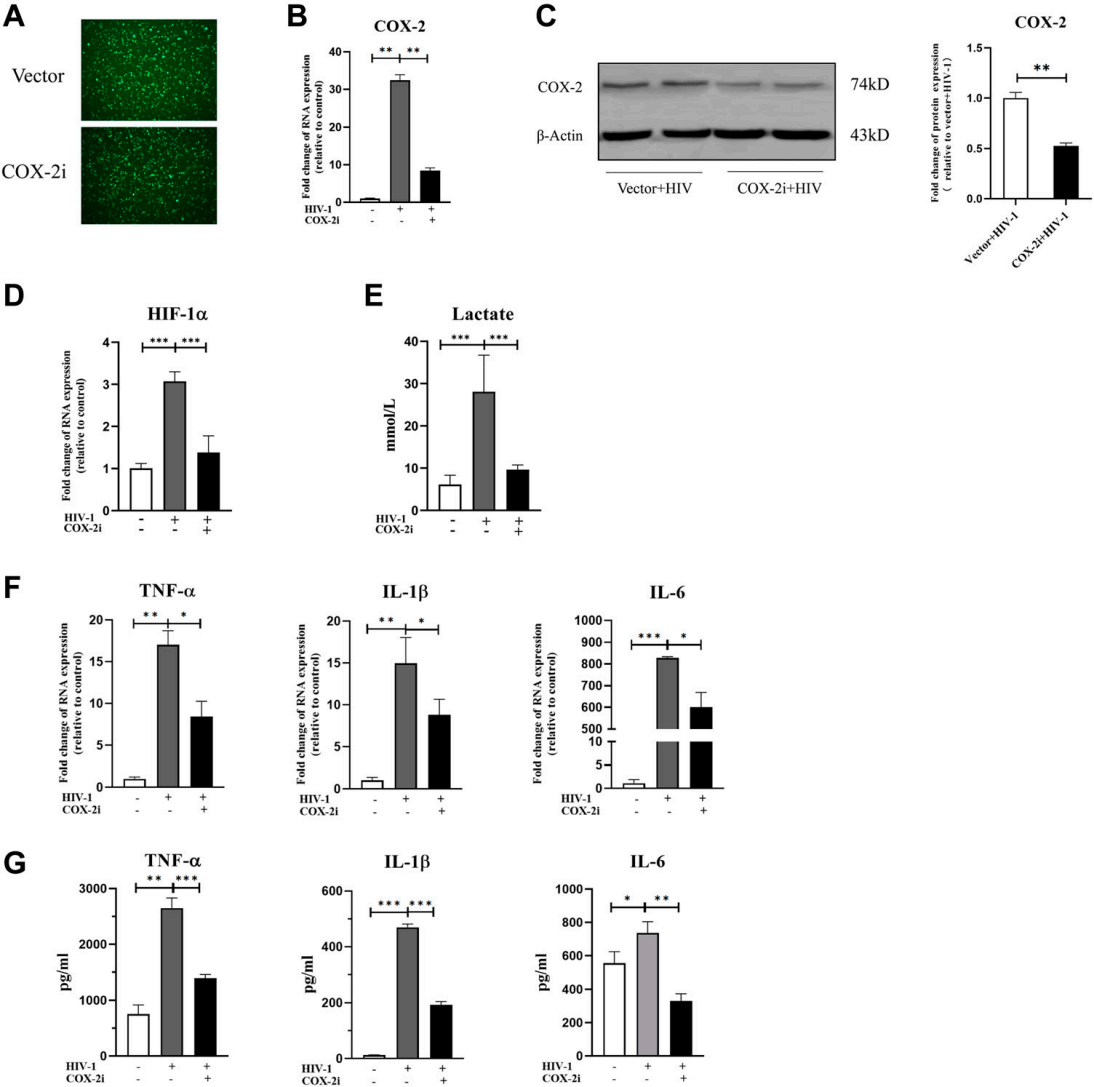
Knockdown of COX-2 represses HIF-1ɑ–dependent glycolytic shift and M1 polarization in THP-1 macrophages. **(A)** EGFP fluorescence of THP-1 macrophages transfected with lentivirus. **(B, C)** COX-2–silencing THP-1 macrophages were verified by RT–qPCR and Western blot. (Statistical analysis was performed using a *t* test, **P < 0.01.) **(D, E, F, G)** HIF-1ɑ expression (D), lactate production (E), and mRNA and protein expressions of TNF-α, IL-1β, and IL-6 **(F, G)** were decreased in COX-2–silencing THP-1 macrophages, as quantified by RT–qPCR and ELISA. (Statistical analysis was performed using a *t* test, *P < 0.05, **P < 0.01, and ***P < 0.001).

To revalidate the role of COX-2 in regulating M1 polarization of macrophages, we constructed a COX-2–silenced THP-1 cell line using lentivirus-mediated RNAi (Fig 6A). Real-time quantitative PCR (RT-qPCR) and Western blot proved stable COX-2 knockdown expression in THP-1–derived macrophages (COX-2i group) (Fig 6B and C). In COX-2i cells, HIF-1α–dependent glycolysis and the expression of TNF-α, IL-1β, and IL-6 induced by HIV-1 were both reduced (Fig 6D–G), which was consistent with the results obtained from meloxicam treatment. These findings indicated that COX-2 plays an important role in mediating HIF-1α–dependent immunometabolic reprogramming of macrophages upon HIV-1 infection.

### Phosphorylation of JNK involves in HIV-1–regulating immunometabolic reprogramming in macrophages

**Figure 7.**
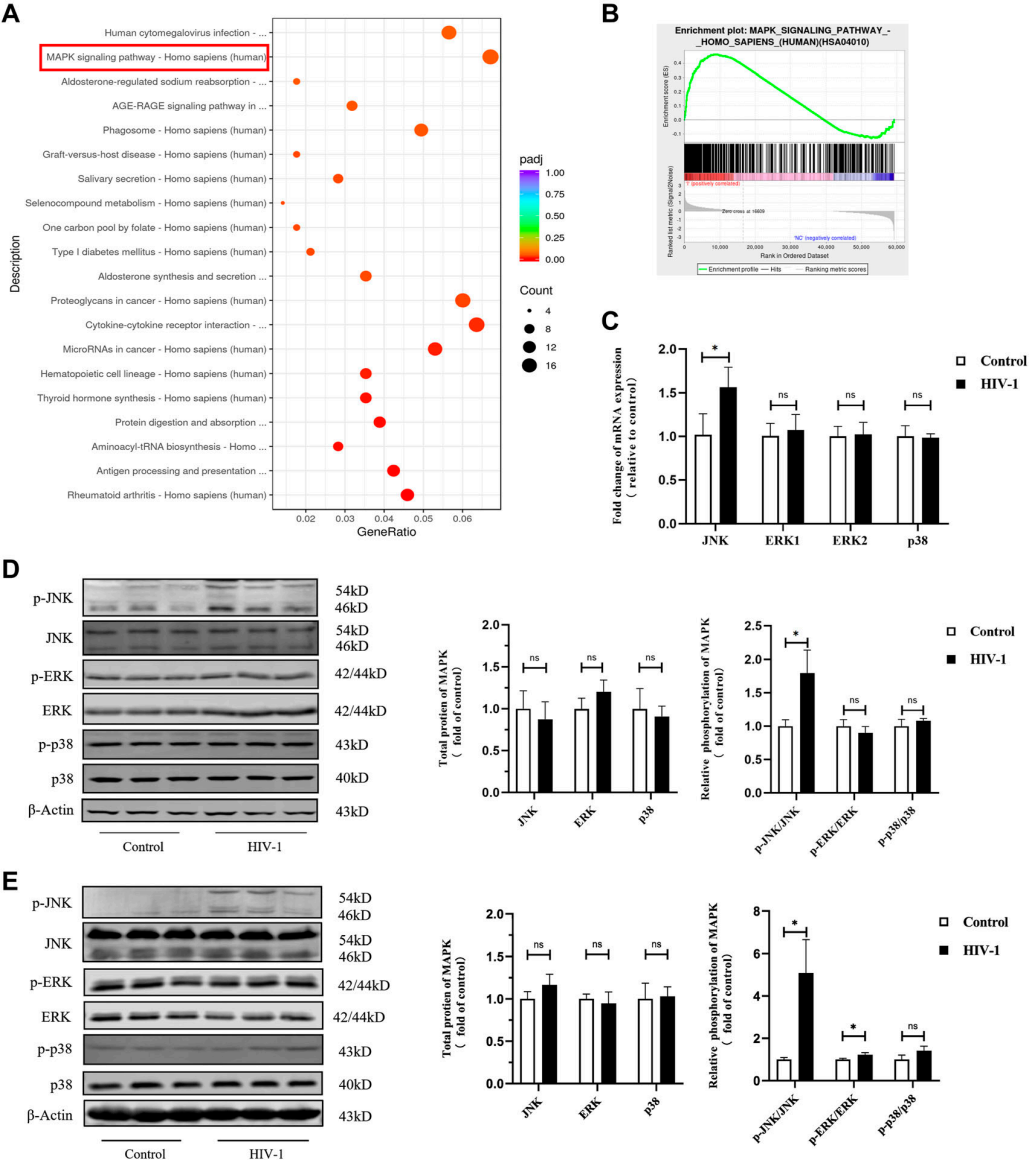
HIV-1 urges phosphorylation of JNK in macrophages. THP-1 macrophages and primary monocyte-derived macrophages (MDMs) were infected with or without HIV-1 for 48 h. **(A)** KEGG enrichment analysis of differentially expressed genes in HIV-1–infected THP-1 macrophages and control macrophages. The size of the bubble positively correlated with the number of enriched genes. The x-axis represents the gene ratio, and the color of the bubble represents the adjusted *P*-value of enrichment analysis. **(B)** Gene set enrichment analysis was performed in Control and HIV-1 groups. In this figure, the y-axis represents enrichment score (ES), and on the x-axis are genes (vertical black lines) included in gene sets. The analysis demonstrates that the MAPK signaling pathway is enriched in the HIV-1 group (NES = 1.315, *P*-value < 0.0001, FDR = 0.301). The detailed information is provided in Table S1. **(C)** RT–qPCR analysis showed the mRNA levels of JNK, ERK, and p38 in MDMs. (Statistical analysis was performed using a *t* test, *P < 0.05.) **(D, E)** Western blot confirmed phosphorylation of JNK is increased in MDMs (D) and THP-1 macrophages (E) upon HIV-1 infection. (Statistical analysis was performed using a *t* test, *P < 0.05).

To further explore the upstream events of COX-2 activation, we analyzed the differential genes between HIV-1–infected macrophages and control macrophages using bioinformatics techniques. Based on our transcriptome sequencing (RNA-seq) data, we found the MAPK signal transduction pathway was significantly enriched (Fig 7A). Gene set enrichment analyses revealed that genes involved in the MAPK pathway were more expressed in the HIV-1 infection group than those in the control group (Fig 7B, Table S1), supporting that MAPK signaling is associated with response to HIV-1 infection in macrophages. To further investigate which subpathway response is more sensitive to HIV-1 infection, we measured the expression of JNK, ERK1/2, and p38. At the mRNA level, we found that only JNK expression in the infection group was higher than that in the control group (*P* < 0.05), but there was no significant difference in the expression of total JNK at the protein level (Fig 7C–E). However, at the protein phosphorylation level, we found the ratio of p-JNK to total JNK protein was significantly higher in the HIV-1 infection group compared with the control group, whereas the ratios of p-ERK to ERK and of p-p38 to p38 had no significant differences between two groups (Fig 7C–E). Therefore, it is plausible that HIV-1 causes immune response of macrophages via activating the MAPK/JNK signaling pathway.

Table S1. Gene set enrichment analysis of the MAPK signaling pathway.

**Figure 8.**
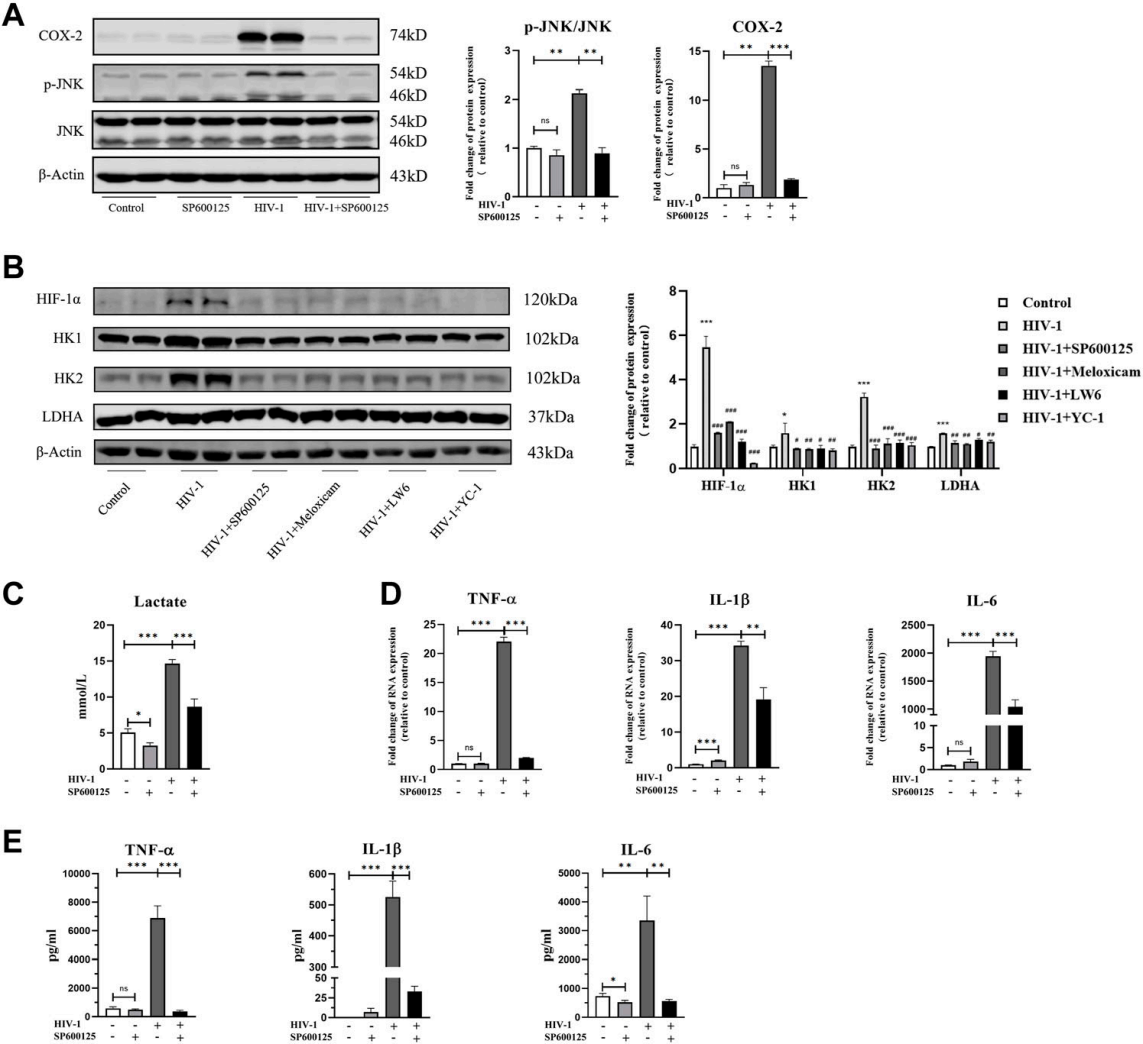
SP600125 reverses HIV-1–induced M1 polarization by inhibiting JNK activation. Monocyte-derived macrophages (MDMs) were pretreated with or without SP600125 (50 μM) for 2 h, followed by infection of HIV-1 for 48 h. **(A)** Expressions of JNK and COX-2 were measured by Western blot. Total JNK was used as the normalization control of phosphorylated JNK, and β-actin was used as the normalization control of COX-2. (Statistical analysis was performed using a *t* test, *P < 0.05, **P < 0.01, and ***P < 0.001.) **(B)** Western blot analysis of hypoxia-inducible factor 1α, HK1, HK2, and LDHA in MDMs. β-Actin was used as the normalization control. SP600125, meloxicam, LW6, and YC-1 were administered as mentioned above. (Statistical analysis was performed using a *t* test, *P < 0.05 and **P < 0.01 as compared to Control; #*P* < 0.05, ##*P* < 0.01, and ###*P* < 0.001 as compared to HIV-1.) **(C)** Lactate concentration in the culture supernatant of MDMs was detected using colorimetry. (Statistical analysis was performed using a *t* test, *P < 0.05 and ***P < 0.001.) **(D, E)** MDMs were collected, and the expression of TNF-α, IL-1β, and IL-6 was measured by RT–qPCR (D) and ELISA (E). (Statistical analysis was performed using a *t* test, *P < 0.05, **P < 0.01, and ***P < 0.001).

We then used SP600125, an inhibitor of JNK, to assess the role of JNK in HIV-1–induced immunometabolic reprogramming in macrophages. In the presence of SP600125 (50 μM), HIV-1–induced phosphorylation of JNK (p-JNK/total JNK) was reduced to the control level (uninfected with HIV-1 and untreated with SP600125), along with a dramatic reduction of HIV-1–induced COX-2 overexpression (Fig 8A), demonstrating the ability of JNK in regulating COX-2 expression. Meanwhile, under inhibition of JNK phosphorylation, the expression of key factors in glycolysis was significantly decreased (Fig 8B). In addition, to verify the role of COX-2 and HIF-1α in p-JNK–mediating metabolic reprogramming and M1 polarization, we also used meloxicam (50 μM), LW6 (15 μM), and YC-1 (4 μM) to specifically inhibit the COX-2 pathway and HIF-1α pathway, respectively. The results confirmed the inhibition of COX-2 or HIF-1α led to the decreased expression of key factors (HIF-1α, HK1, HK2, and LDHA) in glycolysis (Fig 8B), which provided evidence at the protein level to support previous results in Figs 3 and 5. As a result of p-JNK inhibition, the HIV-1–induced lactate displayed a 30–50% reduction compared with HIV-1 infection alone, indicating the weakened glycolysis (Fig 8C). Furthermore, the inhibition of p-JNK, along with the suppression of COX-2 and HIF-1α, as well as the inhibition of glycolysis, resulted in decreased M1 polarization. Most notably, expressions of TNF-α and IL-6 were not increased under SP600125 treatment, despite the strong M1 induction from HIV-1 demonstrated above (Fig 8D and E).

Taken together, by analyzing changes in COX-2 expression, glycolysis level, and M1 polarization in the presence or absence of JNK inhibition, we found that phosphorylation of JNK promotes glycolysis enhancing, which is regulated by the COX-2/HIF-1α signaling axis, supporting M1 polarization of macrophages in HIV-1 infection.

## Discussion

In this study, using in vitro models of HIV-1–infected MDMs and THP-1 macrophages, we observed that macrophages were mainly driven to M1-like changes, with a large amount of pro-inflammatory cytokines produced, whereas the expression of M2-related markers was slightly changed. This is largely consistent with previous reports in vivo or in vitro (Porcheray et al, 2006; Cassol et al, 2010; Burrack & Morrison, 2014), showing that macrophages tend to be M1-polarized during HIV-1 infection. The current understanding of M1 polarization in macrophages is mainly based on studies of LPS and IFN-γ stimulation. It has been found that JAK/STAT (Runtsch et al, 2022), PI3K/Akt (Vergadi et al, 2017), or Notch (Lin et al, 2018) signaling pathways are involved in polarization of macrophages under different stimuli or different microenvironments (Yunna et al, 2020). However, the molecular mechanism by which HIV-1 regulates macrophage polarization is not fully understood.

Immune cells adopt diverse metabolic strategies for different activation states, which are essential to resist pathogen invasion and maintain microenvironmental homeostasis (Russell et al, 2019). For example, activated T cells promote glycolysis and production of proteins, as well as nucleic acids, whereas Tregs and memory T cells rely on oxidization of fatty acids for fuel (Maciolek et al, 2014). For macrophages, evidence suggests that IFN-γ– or other pro-inflammatory factor–induced M1 macrophages use the glycolysis pathway and the pentose phosphate pathway as energy sources, whereas TCA, OXPHOS, and FAO are down-regulated. In contrast, M2 macrophages increase FAO, glutaminolysis, and mitochondrial respiration (Saha et al, 2017; Viola et al, 2019). In the context of HIV infection, Palmer and co-workers have revealed that CD4^+^ T cells activated by HIV-1 infection switch metabolic phenotype from oxidative metabolism to aerobic glycolysis (Palmer et al, 2014). In addition, another study showed that central memory HIV-1–specific CD8^+^ T cells have a metabolic profile characterized by elevated glycolysis with activation of the mTORC1 pathway (Angin et al, 2019). On the contrary, it has been shown that OXPHOS of primary myeloid dendritic cells (mDCs) from elite controllers (ECs) with very low levels of HIV-1 infection is increased compared with both HIV-1–positive individuals undergoing combination antiretroviral therapy and healthy individuals (Hartana et al, 2021). In this study, we turn our attention to the changes in glucose metabolism in macrophages upon HIV-1 infection. By measuring glucose uptake, lactate concentration, OCR, and ECR, we found enhanced glycolysis and impaired TCA and OXPHOS in HIV-1–infected macrophages, which are similar to those observed in T cells by Palmer (Palmer et al, 2014). However, there are some articles that take different views. For example, Castellano et al found that HIV-1 infection of macrophages leads to lipid accumulation and TCA regulation disorder, but does not cause changes in glycolysis (Castellano et al, 2019). This discrepancy may be due to the different cell culture protocols. The usage of M-CSF in the culture of macrophages in Castellano’s study could promote M2 polarization (Chen et al, 2021), which is accompanied by a different metabolic profile from that of M1 macrophages (Na et al, 2015). To avoid the influence of M-CSF on macrophage metabolism, in our study, the primary macrophages were differentiated without additional cytokine stimulation, as some previous studies described (Sharma et al, 2022).

**Figure S3.**
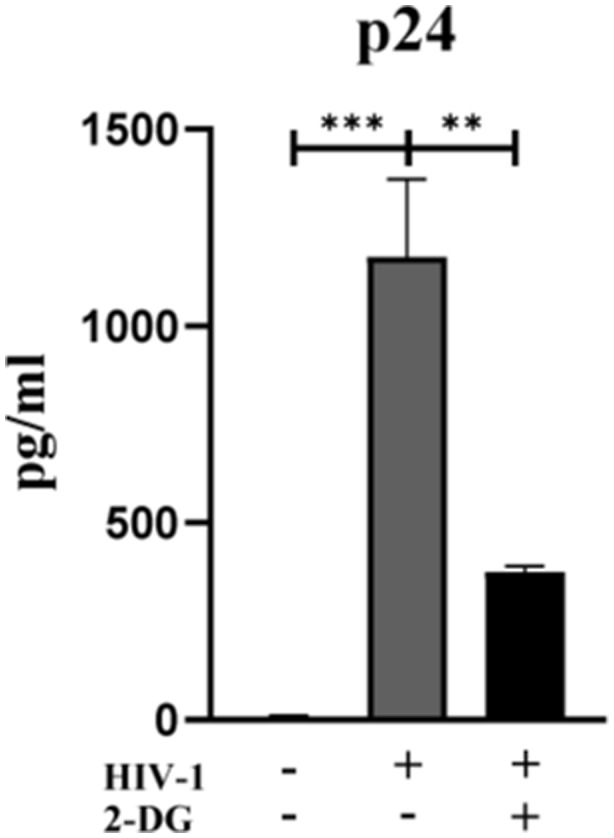
Blockade of glycolysis inhibits HIV-1 replication. Monocyte-derived macrophages were pretreated with or without 2-DG (10 mM) for 2 h, followed by infection of HIV-1 for 48 h. The content of p24 protein in the supernatant was detected by ELISA. (Statistical analysis was performed using a *t* test, *P < 0.05, **P < 0.01, and ***P < 0.001).

Macrophage M1 polarization, accompanied by the release of a large number of pro-inflammatory cytokines, is an important source of chronic inflammation in HIV-1–infected patients. Currently, the use of glucose to locate inflamed tissues has been clinically applied, such as ^18^F-fluorodeoxyglucose–positron emission tomography (^18^F-FDG-PET) imaging (Rudd et al, 2002; Sarrazin et al, 2012). Hammoud et al performed ^18^F-FDG-PET in HIV patients and found those with high levels of glycolysis in CD4^+^ T cells and monocytes before ART initiation were more likely to develop immune reconstitution inflammatory syndrome (Hammoud et al, 2019). This suggests that increased glycolysis may be related to the inflammatory status in HIV-1–infected patients. In the present study, by blocking glycolysis with 2-DG or heptelidic acid, we observed reversal of the increased pro-inflammatory cytokines induced by HIV-1 infection, indicating that macrophages prime glycolytic shift to facilitate M1 polarization in HIV-1 infection, further pointing to the clinical potential of reducing inflammation in HIV-1–infected patients by correcting glucose metabolism. It is worth noting that glucose metabolic regulation of immune cells can also influence HIV-1 pathogenesis. The study of CD4^+^ T cells has shown that HIV-1 infection is strongly impaired when glucose metabolism is inhibited (Valle-Casuso et al, 2019). Consistent with that, in this study, HIV-1–infected macrophages showed a significant increase in ATP production, particularly from glycolysis. After inhibiting glycolysis, we found that HIV-1 replication was reduced (Fig S3). Enhanced glucose metabolism favors HIV-1 infection, perhaps because metabolically active cells have increased the susceptibility to HIV-1 infection. Another possibility is that metabolic activation produces molecules necessary for HIV-1 survival (Sáez-Cirión et al, 2021).

In the present study, we found that HIF-1α was markedly up-regulated by HIV-1 infection, and inhibition of HIF-1α reduced HIV-1–induced glycolysis and M1 polarization. Activation of glycolysis by HIF-1α is considered critical for immunometabolic adaptation in monocytes (Cheng et al, 2014; Kim et al, 2006). As a transcription factor, HIF-1α mediates the expression of glycolytic enzymes (Semenza et al, 1994). Previous studies have revealed that HK is up-regulated in HIV-1–infected CD4^+^ T cells (Kavanagh Williamson et al, 2018). Consistently, we observed glycolytic enzymes such as HK1/2, PDK1, and LDHA were up-regulated in HIV-1–infected macrophages, whereas inhibition of HIF-1α repressed the expression of glycolytic enzymes. The evidence suggests that HIV-1–manipulated immunometabolism may be achieved through the regulation of multiple glycolytic enzymes, and HIF-1α serves as the pivotal mediator in this progress. Because HIF-1α activation has also been reported to suppress mitochondrial function in cancer (Semenza, 2007; Nagao et al, 2019), we speculate that the deceased mitochondrial respiration in HIV-1–infected macrophages may be regulated by HIF-1α. We then tried two inhibitors of HIF-1α; however, neither of them recovered the attenuated OXPHOS. In fact, in peritoneal macrophages derived from Hif-1α ^−/−^ mice, the level of OXPHOS was also not elevated (Yu et al, 2020). Our results, combined with previous studies, suggest that the regulatory mechanism of OXPHOS in macrophages is specific to different tumor microenvironments or pathogen invasions. Therefore, HIV-1–mediated mitochondrial damage in macrophages was HIF-1α–independent, and other mechanisms are worthy of further investigation.

We further explored the upstream events of HIV-1–induced HIF-1α expression and found that COX-2 was significantly up-regulated in macrophages when exposed to HIV-1. COX-2 is rarely expressed in steady-state cells, whereas it is boosted in activated cells (O’Banion, 1999). Traditionally, COX-2 mediates inflammatory response through PGE2 production (Mizuno et al, 2019). Interestingly, recent studies have found that in addition to PGE2, COX-2 can also regulate the HIF-1α/PKM2 pathway in apoptosis resistance (Wang et al, 2019). In HIV-1–induced M1 polarization, we observed that the activation of HIF-1α, enhancement of glycolysis, and pro-inflammatory cytokine secretion were effectively reversed under COX-2 inhibition, indicating that HIV-1 promotes HIF-1α–dependent glycolysis and M1 polarization through COX-2.

As one of the central pathways in various cellular programs, the MAPK signaling pathway has been demonstrated to participate in immune regulation through COX-2 (Cargnello & Roux, 2011; Lin et al, 2015). Here, we investigated the role of phosphorylation of JNK, ERK, or p38 in regulation of COX-2 by HIV-1, and found that HIV-1 promoted phosphorylation of JNK, but not ERK and p38. In addition, we confirmed that COX-2 was positively regulated by JNK phosphorylation in the context of HIV-1 infection. Currently, the role of the MAPK signaling pathway in glucose metabolism remains controversial. For instance, MAPKs increase glucose uptake during exercise, whereas they mediate glucose intolerance in metabolic syndrome (Schultze et al, 2012; Bengal et al, 2020). In this study, inhibition of JNK suppressed HIF-1α–dependent glycolysis and reduced secretion of pro-inflammatory factors, supporting that HIV-1–induced immunometabolic reprogramming in macrophages is modulated by JNK and its downstream COX-2 activation.

In summary, the present study suggests that M1 polarization of macrophages is related to glycolysis promotion via the JNK/COX-2/HIF-1α signaling axis in HIV-1 infection. The findings broaden our understanding of mechanisms underlying macrophage polarization, and imply that alleviating glycolysis may be a strategy to reduce inflammatory levels in individuals living with HIV-1.

## Materials and Methods

### Ethics statement

Guangxi Medical University Ethical Committee approved all experimental procedures and protocols used in this study (Approval No. 2022-0130).

### Reagents and antibodies

PMA, LPS, IFN-γ, IL-4, IL-10, and SP600125 were purchased from Sigma-Aldrich. 2-Deoxy-D-glucose (2-DG), heptelidic acid (HA), oligomycin (Omy), LW6, and YC-1 were from MCE (United States). Meloxicam was from TCI (Japan). Immunofluorescence blocking buffer (#12411), and Alexa Fluor 488 goat anti-rabbit (#4412; RRID: AB_1904025) and Alexa Fluor 594 goat anti-mouse (#8890; RRID: AB_2714182) secondary antibodies were purchased from Cell Signaling Technology. Antibodies against β-actin (#3700; RRID: AB_2242334), phospho-SAPK/JNK (#4668; RRID: AB_823588), SAPK/JNK (#9252; RRID: AB_2250373), phospho-p44/42 MAPK (Erk1/2) (#4370; RRID: AB_2315112), p44/42 MAPK (Erk1/2) (#4695; RRID: AB_390779), phospho-p38 MAPK (#4511; RRID: AB_2139682), p38 MAPK (#8690; RRID: AB_10999090), COX-2 (#12282; RRID: AB_2571729), and HIF-1α (#36169; RRID: AB_2799095) and Glycolysis Antibody Sampler Kit (#8337; RRID: AB_10897509) were obtained from Cell Signaling Technology. The secondary antibodies of IRDye 680RD donkey anti-mouse IgG and IRDye 800CW donkey anti-rabbit IgG were purchased from LI-COR.

### Cell culture and differentiation

The human monocyte THP-1 cell line (conserved in our laboratory, ATCC number: TIB-202; RRID: CVCL_0006) and primary human macrophages were used as cell models for HIV-1 infection. THP-1 cells were routinely cultured in 1640 medium (Gibco) containing 10% heat-inactivated FBS (Gibco) and 1% penicillin–streptomycin solution (Solarbio) at 37°C and 5% CO_2_ atmosphere. To obtain THP-1–derived macrophages, THP-1 cells were seeded in a six-well culture plate (10^6^ cells per well) with DMEM (Gibco) containing 10% FBS and 1% penicillin–streptomycin solution, and stimulated with 50 ng/ml of PMA (48 h) for differentiation (Dunn et al, 2011). Primary human macrophages were derived from PBMCs of healthy donors as previously described (Fang et al, 2009). Briefly, PBMCs were isolated from whole blood by Ficoll (GE Healthcare) through density gradient centrifugation according to the manufacturer’s protocol (Riedhammer et al, 2016). PBMCs were then incubated in 75-cm^2^ flasks precoated with gelatin (VETEC) in a 37°C, 5% CO_2_ atmosphere for 30 min. Adhesive monocytes were harvested using EDTA (Solarbio). Collected monocytes were seeded at a density of 4 × 10^6^ cells/ml in complete 1,640 medium (containing 10% human A^+^B^+^ serum) and refreshed medium every 3 d until differentiation into macrophages for experiments.

### Virus production and infection

HIV-1_Bal_ virions were originally from the NIH AIDS Reagent Program and reproduced in our laboratory. The modified method was based on Edward’s report (Barker et al, 1998). In brief, PHA (5 μg/ml) in complete 1,640 medium (containing 100 U/ml IL-2) stimulated human PBMCs for 3 d at 37°C, 5% CO_2_. Afterward, polybrene (2 μg/ml) was added to enhance HIV-1 infection. After HIV-1_Bal_ infection, the supernatant was harvested every 3 d and viral titers were quantified using a HIV-1 p24 ELISA kit (Jianglaibio). We adjusted the viral titer to 100 ng p24/ml and stored it at −80°C.

HIV-1 infection was performed after differentiation of monocytes into macrophages. HIV-1 virions were added to culture medium (8 ng p24/10^6^ cells) for 6 h. Then, cells were washed with PBS (Solarbio) and cultured in fresh medium until harvest at the appointed time.

### Lentiviral construction and transduction

Lentiviral particles were obtained from GeneChem. In detail, the siRNAs for COX-2 (NM_000963.4) (COX-2i) were chimeric into the GV248 plasmid. The sequence for the Vector was as follows: 5′-TTCTCCGAACGTGTCACGT-3′. The sequence for COX-2i was as follows: 5′-GCTGAATTTAACACCCTCTAT-3′. Titers of concentrated viral particles were 8.0 × 10^8^ units/ml. Lentiviral particles were transduced into THP-1 cells during the logarithmic growth phase. The supernatant was removed after 24 h of infection and replaced with complete 1,640 medium. To generate stable COX-2 knockdown cell lines, 2 μg/ml puromycin was added to medium for 48 h. A fluorescent microscope was used to observe successful transduction. RT-qPCR and Western blot were used to confirm the down-regulated expression of COX-2 in COX-2i THP-1 cells compared with Vector THP-1 cells.

### RNA extraction and RT-qPCR

Total cellular RNA was extracted using TRIzol (Sigma-Aldrich). The cells were pipetted with TRIzol (40 μl/10^5^ cells) until completely lysed, and chloroform (Tedia) was added for stratification by the gradient. After centrifugation (4°C, 12,000 g/min, 15 min), the transparent supernatant was collected. Isopropanol (Tedia) was used to precipitate RNA, and 75% ethanol was for purification. The remaining RNA pellet was resuspended in enzyme-free water, followed by incubation at 55°C for 15 min. The quantification was performed using NanoDrop 2000C (United States). The cDNA was synthesized with PrimeScript RT Master Mix (Takara) using 1 μg RNA. cDNA samples were used for RT-qPCR with SYBR Premix Ex Taq^TM^ (Takara) according to the operation manual. The sequences of primer pairs are listed in Table S2. Relative mRNA expressions were analyzed based on the 2^−ΔΔCt^ method, normalizing to the housekeeping gene GAPDH.

Table S2. Primer list.

### Cytokine and lactate measurements

Culture supernatants were collected at the indicated time points, and protein concentrations of TNF-α, IL-1β, and IL-6 were quantified by ELISA according to the manufacturer’s instructions (NOVUS). Lactate production was measured by a lactate kit (Njjcbio) using colorimetry according to the manufacturer’s instructions.

### Flow cytometry

For detection of polarization markers of macrophages, MDMs were harvested by scraping. The harvested cells were washed with PBS to remove the culture medium thoroughly and collected by centrifugation. For intracellular staining, the cells were fixated and permeabilized using the Cytofix/Cytoperm kit (BD Biosciences), then stained with PE-linked anti-CD68 (#12-0689-41, RRID: AB_10805746; eBioscience), APC-linked anti-CD86 (#17-0869-41, RRID: AB_2802218; eBioscience), and APC-linked anti-CD206 (# 17-2069-42, RRID: AB_2573182; eBioscience) for 20 min at 4°C. The stained cells were washed with PBS and finally resuspended in PBS. Flow cytometry was performed using a CytoFLEX2 flow cytometer (Beckman Coulter) and analyzed by FlowJo software.

### Western blot

Protein samples were prepared using lysis buffer (RIPA: PMSF = 100:1, RIPA: phosphatase inhibitors = 100:1). The total protein concentration of samples was quantified using the BCA Protein Assay kit (Beyotime) to make sure each sample had an equal amount of protein for SDS–PAGE. After transferring to PVDF membranes, the membranes were blocked with 5% skim milk for 1 h at RT and then incubated with primary antibody overnight at 4°C. Fluorescent secondary antibody was used for detection. Relative band intensities were measured by the LI-COR Odyssey CLx imaging system.

### ATP assay and ATP/ADP ratio assay

ATP concentrations were measured using CellTiter-Glo Kit (Promega) following the manufacturer’s protocol. Differentiated macrophages were infected with or without HIV-1 for 48 h in refreshed medium, and incubated with detection reagents at 37°C for 10 min. The fluorescence intensity was read under a microplate reader (Synergy H1; BioTek). ATP concentrations were determined and normalized to the protein level of each sample.

Changes in the ATP/ADP ratio were detected using ATP/ADP-Lite Assay Kit (Vigorous Bio). The treated MDMs were lysed with lysis buffer for 10 min at RT with gentle shaking. Subsequently, 50 μl of assay reagent was mixed with 10 μl of the cell lysate, and the samples were then analyzed using a microplate reader to obtain the initial readings (Data A). After a 10-min incubation, another reading was taken (Data B). To each sample, 10 μl of ATP converting enzyme was added, followed by another reading after 10 min (Data C). The ATP/ADP ratio was calculated using the following formula:

ATP/ ADP ratio=Data A/(Data C−Data B)

### Glucose uptake assay

Glucose uptake was measured using 2-NBDG Glucose Uptake Assay Kit (BioVision) according to the manufacturer’s instructions. Briefly, MDMs (10^5^ cells) were seeded in a 24-well plate with 400 μl detection reagent (376 μl 0.5% serum 1,640 cell culture medium, 4 μl 2-NBDG reagent, and 20 μl glucose uptake enhancer) at 37°C for 30 min. Subsequently, cells were washed with iced analysis buffer three times and scraped off with rubber policeman. Fluorescence intensity was examined by a Beckman flow cytometer (excitation at 488 nm).

### Extracellular oxygen consumption and acidification test

Changes in oxygen consumption and acidification after HIV-1 infection were analyzed using assays from Abcam (#ab197243 and #ab197244). Macrophages were seeded in a 96-well plate (black wall) at a density of 10^5^ cells/well. The cell culture medium was replaced with an extracellular oxygen consumption reagent or acidification assay reagent, and fluorescence was monitored using the time-resolved fluorescence (Synergy H1; BioTek). Results were normalized to the total DNA content, which was assessed with Hoechst dye (R&D).

### MMP detection

MMP was measured by JC-1 (Beyotime). MDMs were seeded in a 48-well plate. JC-1 dye was prepared according to the instructions and added to the plate for incubation at 37°C for 20 min. The cells were then washed with iced washing buffer. Images were acquired using a fluorescence microscope, and image processing was performed using ImageJ software.

### Immunofluorescence staining

MDMs were prepared for immunofluorescence staining. 4% formaldehyde and 100% cold methanol were used for fixation and permeation, respectively. Blocking was performed with blocking buffer at RT for 60 min. Cells were then incubated with rabbit anti-HIF-1α (1:500) or anti-β-actin (1:5,000) overnight at 4°C. The immune complexes were glowed using Alexa Fluor 488 goat anti-rabbit (1:1,000) or Alexa Fluor 594 goat anti-mouse (1:1,000) secondary antibodies before counterstaining with DAPI (Solarbio). Images were obtained using a fluorescence microscope (EVOS FL Auto2; Invitrogen), and image processing was performed using ImageJ software.

### Statistical analysis

All experiments were repeated at least three times. Statistical analysis was performed using a *t* test with SPSS v25.0 and GraphPad Prism v8.0. Data were presented as the mean ± SD, and *P* < 0.05 was considered as the significance of differences between the two groups.

## Data Availability

All data are available in the article or the supplementary materials. Raw data generated during the current study will be available from the corresponding author on reasonable request.

## Supplementary Material

Reviewer comments

## References

[bib1] Angin M, Volant S, Passaes C, Lecuroux C, Monceaux V, Dillies MA, Valle-Casuso JC, Pancino G, Vaslin B, Le Grand R, (2019) Metabolic plasticity of HIV-specific CD8(+) T cells is associated with enhanced antiviral potential and natural control of HIV-1 infection. Nat Metab 1: 704–716. 10.1038/s42255-019-0081-4 32694646

[bib2] Barker E, Mackewicz CE, Reyes-Terán G, Sato A, Stranford SA, Fujimura SH, Christopherson C, Chang SY, Levy JA (1998) Virological and immunological features of long-term human immunodeficiency virus-infected individuals who have remained asymptomatic compared with those who have progressed to acquired immunodeficiency syndrome. Blood 92: 3105–3114. 10.1182/blood.v92.9.3105 9787145

[bib3] Bengal E, Aviram S, Hayek T (2020) p38 MAPK in glucose metabolism of skeletal muscle: Beneficial or harmful? Int J Mol Sci 21: 6480. 10.3390/ijms21186480 32899870 PMC7555282

[bib4] Burrack KS, Morrison TE (2014) The role of myeloid cell activation and arginine metabolism in the pathogenesis of virus-induced diseases. Front Immunol 5: 428. 10.3389/fimmu.2014.00428 25250029 PMC4157561

[bib5] Cargnello M, Roux PP (2011) Activation and function of the MAPKs and their substrates, the MAPK-activated protein kinases. Microbiol Mol Biol Rev 75: 50–83. 10.1128/mmbr.00031-10 21372320 PMC3063353

[bib6] Cashin K, Roche M, Sterjovski J, Ellett A, Gray LR, Cunningham AL, Ramsland PA, Churchill MJ, Gorry PR (2011) Alternative coreceptor requirements for efficient CCR5- and CXCR4-mediated HIV-1 entry into macrophages. J Virol 85: 10699–10709. 10.1128/jvi.05510-11 21835796 PMC3187472

[bib7] Cassol E, Cassetta L, Rizzi C, Alfano M, Poli G (2009) M1 and M2a polarization of human monocyte-derived macrophages inhibits HIV-1 replication by distinct mechanisms. J Immunol 182: 6237–6246. 10.4049/jimmunol.0803447 19414777

[bib8] Cassol E, Cassetta L, Alfano M, Poli G (2010) Macrophage polarization and HIV-1 infection. J Leukoc Biol 87: 599–608. 10.1189/jlb.1009673 20042468

[bib9] Castellano P, Prevedel L, Valdebenito S, Eugenin EA (2019) HIV infection and latency induce a unique metabolic signature in human macrophages. Sci Rep 9: 3941. 10.1038/s41598-019-39898-5 30850623 PMC6408492

[bib10] Chen YC, Lai YS, Hsuuw YD, Chang KT (2021) Withholding of M-CSF supplement reprograms macrophages to M2-like via endogenous CSF-1 activation. Int J Mol Sci 22: 3532. 10.3390/ijms22073532 33805444 PMC8037162

[bib11] Cheng SC, Quintin J, Cramer RA, Shepardson KM, Saeed S, Kumar V, Giamarellos-Bourboulis EJ, Martens JH, Rao NA, Aghajanirefah A, (2014) mTOR- and HIF-1α-mediated aerobic glycolysis as metabolic basis for trained immunity. Science 345: 1250684. 10.1126/science.1250684 25258083 PMC4226238

[bib12] Datta PK, Deshmane S, Khalili K, Merali S, Gordon JC, Fecchio C, Barrero CA (2016) Glutamate metabolism in HIV-1 infected macrophages: Role of HIV-1 Vpr. Cell Cycle 15: 2288–2298. 10.1080/15384101.2016.1190054 27245560 PMC5004675

[bib13] Deeks SG, Lewin SR, Havlir DV (2013) The end of AIDS: HIV infection as a chronic disease. Lancet 382: 1525–1533. 10.1016/s0140-6736(13)61809-7 24152939 PMC4058441

[bib14] Dunn WB, Broadhurst D, Begley P, Zelena E, Francis-McIntyre S, Anderson N, Brown M, Knowles JD, Halsall A, Haselden JN, (2011) Procedures for large-scale metabolic profiling of serum and plasma using gas chromatography and liquid chromatography coupled to mass spectrometry. Nat Protoc 6: 1060–1083. 10.1038/nprot.2011.335 21720319

[bib15] Engelhardt G (1996) Pharmacology of meloxicam, a new non-steroidal anti-inflammatory drug with an improved safety profile through preferential inhibition of COX-2. Br J Rheumatol 35: 4–12. 10.1093/rheumatology/35.suppl_1.4 8630636

[bib16] Fang HY, Hughes R, Murdoch C, Coffelt SB, Biswas SK, Harris AL, Johnson RS, Imityaz HZ, Simon MC, Fredlund E, (2009) Hypoxia-inducible factors 1 and 2 are important transcriptional effectors in primary macrophages experiencing hypoxia. Blood 114: 844–859. 10.1182/blood-2008-12-195941 19454749 PMC2882173

[bib17] Hammoud DA, Boulougoura A, Papadakis GZ, Wang J, Dodd LE, Rupert A, Higgins J, Roby G, Metzger D, Laidlaw E, (2019) Increased metabolic activity on 18F-fluorodeoxyglucose positron emission tomography-computed tomography in human immunodeficiency virus-associated immune reconstitution inflammatory syndrome. Clin Infect Dis 68: 229–238. 10.1093/cid/ciy454 30215671 PMC6321853

[bib18] Hartana CA, Rassadkina Y, Gao C, Martin-Gayo E, Walker BD, Lichterfeld M, Yu XG (2021) Long noncoding RNA MIR4435-2HG enhances metabolic function of myeloid dendritic cells from HIV-1 elite controllers. J Clin Invest 131: e146136. 10.1172/jci146136 33938445 PMC8087208

[bib19] Hegedus A, Kavanagh Williamson M, Huthoff H (2014) HIV-1 pathogenicity and virion production are dependent on the metabolic phenotype of activated CD4+ T cells. Retrovirology 11: 98. 10.1186/s12977-014-0098-4 25421745 PMC4252996

[bib20] Hegedus A, Kavanagh Williamson M, Khan MB, Dias Zeidler J, Da Poian AT, El-Bacha T, Struys EA, Huthoff H (2017) Evidence for altered glutamine metabolism in human immunodeficiency virus type 1 infected primary human CD4(+) T cells. AIDS Res Hum Retroviruses 33: 1236–1247. 10.1089/aid.2017.0165 28844150 PMC5709700

[bib21] Kavanagh Williamson M, Juszczak F, Athanasopoulos M, Khan MB, Eykyn TR, Srenathan U, Taams LS, Zeidler JD, Da Poian AT, Huthoff H, (2018) Upregulation of glucose uptake and hexokinase activity of primary human CD4+ T cells in response to infection with HIV-1. Viruses 10: 114. 10.3390/v10030114 29518929 PMC5869507

[bib22] Kim JW, Tchernyshyov I, Semenza GL, Dang CV (2006) HIF-1-mediated expression of pyruvate dehydrogenase kinase: A metabolic switch required for cellular adaptation to hypoxia. Cell Metab 3: 177–185. 10.1016/j.cmet.2006.02.002 16517405

[bib23] Kim Y, Nurakhayev S, Nurkesh A, Zharkinbekov Z, Saparov A (2021) Macrophage polarization in cardiac tissue repair following myocardial infarction. Int J Mol Sci 22: 2715. 10.3390/ijms22052715 33800220 PMC7962533

[bib24] Lin CK, Tseng CK, Chen KH, Wu SH, Liaw CC, Lee JC (2015) Betulinic acid exerts anti-hepatitis C virus activity via the suppression of NF-κB- and MAPK-ERK1/2-mediated COX-2 expression. Br J Pharmacol 172: 4481–4492. 10.1111/bph.13233 26102077 PMC4562509

[bib25] Lin Y, Zhao JL, Zheng QJ, Jiang X, Tian J, Liang SQ, Guo HW, Qin HY, Liang YM, Han H (2018) Notch signaling modulates macrophage polarization and phagocytosis through direct suppression of signal regulatory protein α expression. Front Immunol 9: 1744. 10.3389/fimmu.2018.01744 30105024 PMC6077186

[bib26] Maciolek JA, Pasternak JA, Wilson HL (2014) Metabolism of activated T lymphocytes. Curr Opin Immunol 27: 60–74. 10.1016/j.coi.2014.01.006 24556090

[bib27] Maldonado EN, Lemasters JJ (2014) ATP/ADP ratio, the missed connection between mitochondria and the Warburg effect. Mitochondrion 19 PA: 78–84. 10.1016/j.mito.2014.09.002 25229666 PMC4254332

[bib28] Mizuno R, Kawada K, Sakai Y (2019) Prostaglandin E2/EP signaling in the tumor microenvironment of colorectal cancer. Int J Mol Sci 20: 6254. 10.3390/ijms20246254 31835815 PMC6940958

[bib29] Na YR, Hong JH, Lee MY, Jung JH, Jung D, Kim YW, Son D, Choi M, Kim KP, Seok SH 2nd (2015) Proteomic analysis reveals distinct metabolic differences between granulocyte-macrophage colony stimulating factor (GM-CSF) and macrophage colony stimulating factor (M-CSF) grown macrophages derived from murine bone marrow cells. Mol Cell Proteomics 14: 2722–2732. 10.1074/mcp.M115.048744 26229149 PMC4597147

[bib30] Nagao A, Kobayashi M, Koyasu S, Chow CCT, Harada H (2019) HIF-1-dependent reprogramming of glucose metabolic pathway of cancer cells and its therapeutic significance. Int J Mol Sci 20: 238. 10.3390/ijms20020238 30634433 PMC6359724

[bib31] Nieminen AL, Saylor AK, Herman B, Lemasters JJ (1994) ATP depletion rather than mitochondrial depolarization mediates hepatocyte killing after metabolic inhibition. Am J Physiol 267: C67–C74. 10.1152/ajpcell.1994.267.1.C67 8048493

[bib32] O’Banion MK (1999) Cyclooxygenase-2: Molecular biology, pharmacology, and neurobiology. Crit Rev Neurobiol 13: 45–82. 10.1615/critrevneurobiol.v13.i1.30 10223523

[bib33] O’Brien MP, Hunt PW, Kitch DW, Klingman K, Stein JH, Funderburg NT, Berger JS, Tebas P, Clagett B, Moisi D, (2017) A randomized placebo controlled trial of aspirin effects on immune activation in chronically human immunodeficiency virus-infected adults on virologically suppressive antiretroviral therapy. Open Forum Infect Dis 4: ofw278. 10.1093/ofid/ofw278 28480270 PMC5414028

[bib34] O’Neill LA, Kishton RJ, Rathmell J (2016) A guide to immunometabolism for immunologists. Nat Rev Immunol 16: 553–565. 10.1038/nri.2016.70 27396447 PMC5001910

[bib35] Pajak B, Siwiak E, Sołtyka M, Priebe A, Zieliński R, Fokt I, Ziemniak M, Jaśkiewicz A, Borowski R, Domoradzki T, (2019) 2-Deoxy-d-Glucose and its analogs: From diagnostic to therapeutic agents. Int J Mol Sci 21: 234. 10.3390/ijms21010234 31905745 PMC6982256

[bib36] Palmer CS, Ostrowski M, Gouillou M, Tsai L, Yu D, Zhou J, Henstridge DC, Maisa A, Hearps AC, Lewin SR, (2014) Increased glucose metabolic activity is associated with CD4+ T-cell activation and depletion during chronic HIV infection. Aids 28: 297–309. 10.1097/qad.0000000000000128 24335483 PMC4293200

[bib37] Palsson-McDermott EM, Curtis AM, Goel G, Lauterbach MA, Sheedy FJ, Gleeson LE, van den Bosch MW, Quinn SR, Domingo-Fernandez R, Johnston DG, (2015) Pyruvate kinase M2 regulates Hif-1α activity and IL-1β induction and is a critical determinant of the warburg effect in LPS-activated macrophages. Cell Metab 21: 65–80. 10.1016/j.cmet.2014.12.005 25565206 PMC5198835

[bib38] Perdomo-Celis F, Passaes C, Monceaux V, Volant S, Boufassa F, de Truchis P, Marcou M, Bourdic K, Weiss L, Jung C, (2022) Reprogramming dysfunctional CD8+ T cells to promote properties associated with natural HIV control. J Clin Invest 132: e157549. 10.1172/jci157549 35380989 PMC9151687

[bib39] Porcheray F, Samah B, Léone C, Dereuddre-Bosquet N, Gras G (2006) Macrophage activation and human immunodeficiency virus infection: HIV replication directs macrophages towards a pro-inflammatory phenotype while previous activation modulates macrophage susceptibility to infection and viral production. Virology 349: 112–120. 10.1016/j.virol.2006.02.031 16564558

[bib40] Riedhammer C, Halbritter D, Weissert R (2016) Peripheral blood mononuclear cells: Isolation, freezing, thawing, and culture. Methods Mol Biol 1304: 53–61. 10.1007/7651_2014_99 25092056

[bib41] Rudd JH, Warburton EA, Fryer TD, Jones HA, Clark JC, Antoun N, Johnström P, Davenport AP, Kirkpatrick PJ, Arch BN, (2002) Imaging atherosclerotic plaque inflammation with [18F]-fluorodeoxyglucose positron emission tomography. Circulation 105: 2708–2711. 10.1161/01.cir.0000020548.60110.76 12057982

[bib63] Runtsch MC, Angiari S, Hooftman A, Wadhwa R, Zhang Y, Zheng Y, Spina JS, Ruzek MC, Argiriadi MA, McGettrick AF, (2022) Itaconate and itaconate derivatives target JAK1 to suppress alternative activation of macrophages. Cell Metab 34: 487–501. 10.1016/j.cmet.2022.02.002 35235776

[bib42] Russell DG, Huang L, VanderVen BC (2019) Immunometabolism at the interface between macrophages and pathogens. Nat Rev Immunol 19: 291–304. 10.1038/s41577-019-0124-9 30679807 PMC7032560

[bib43] Sáez-Cirión A, Sereti I (2021) Immunometabolism and HIV-1 pathogenesis: Food for thought. Nat Rev Immunol 21: 5–19. 10.1038/s41577-020-0381-7 32764670

[bib44] Saha S, Shalova IN, Biswas SK (2017) Metabolic regulation of macrophage phenotype and function. Immunol Rev 280: 102–111. 10.1111/imr.12603 29027220

[bib45] Sarrazin JF, Philippon F, Tessier M, Guimond J, Molin F, Champagne J, Nault I, Blier L, Nadeau M, Charbonneau L, (2012) Usefulness of fluorine-18 positron emission tomography/computed tomography for identification of cardiovascular implantable electronic device infections. J Am Coll Cardiol 59: 1616–1625. 10.1016/j.jacc.2011.11.059 22538331

[bib46] Schultze SM, Hemmings BA, Niessen M, Tschopp O (2012) PI3K/AKT, MAPK and AMPK signalling: Protein kinases in glucose homeostasis. Expert Rev Mol Med 14: e1. 10.1017/s1462399411002109 22233681

[bib47] Semenza GL (2007) Oxygen-dependent regulation of mitochondrial respiration by hypoxia-inducible factor 1. Biochem J 405: 1–9. 10.1042/bj20070389 17555402

[bib48] Semenza GL, Roth PH, Fang HM, Wang GL (1994) Transcriptional regulation of genes encoding glycolytic enzymes by hypoxia-inducible factor 1. J Biol Chem 269: 23757–23763. 10.1016/s0021-9258(17)31580-6 8089148

[bib49] Sharma N, Kumar MM, Mohanty KK, Khandelwal V (2022) Optimum use of human AB serum for monocyte-differentiated macrophages (MDMs) isolated from healthy individuals. Re:GEN Open 2: 145–149. 10.1089/regen.2022.0014

[bib50] Simon LS (1999) Role and regulation of cyclooxygenase-2 during inflammation. Am J Med 106: 37s–42s. 10.1016/s0002-9343(99)00115-1 10390126

[bib51] Tan Y, Li J, Zhao G, Huang KC, Cardenas H, Wang Y, Matei DCJX, Cheng JX (2022) Metabolic reprogramming from glycolysis to fatty acid uptake and beta-oxidation in platinum-resistant cancer cells. Nat Commun 13: 4554. 10.1038/s41467-022-32101-w 35931676 PMC9356138

[bib52] Teer E, Joseph DE, Glashoff RH, Faadiel Essop M (2021) Monocyte/macrophage-mediated innate immunity in HIV-1 infection: From early response to late dysregulation and links to cardiovascular diseases onset. Virol Sin 36: 565–576. 10.1007/s12250-020-00332-0 33400091 PMC8379323

[bib53] Utay NS, Overton ET (2020) Immune activation and inflammation in people with human immunodeficiency virus: Challenging targets. J Infect Dis 221: 1567–1570. 10.1093/infdis/jiz351 31282534 PMC7184903

[bib54] Valentín-Guillama G, López S, Kucheryavykh YV, Chorna NE, Pérez J, Ortiz-Rivera J, Inyushin M, Makarov V, Valentín-Acevedo A, Quinones-Hinojosa A, (2018) HIV-1 envelope protein gp120 promotes proliferation and the activation of glycolysis in glioma cell. Cancers (Basel) 10: 301. 10.3390/cancers10090301 30200472 PMC6162763

[bib55] Valle-Casuso JC, Angin M, Volant S, Passaes C, Monceaux V, Mikhailova A, Bourdic K, Avettand-Fenoel V, Boufassa F, Sitbon M, (2019) Cellular metabolism is a major determinant of HIV-1 reservoir seeding in CD4(+) T cells and offers an opportunity to tackle infection. Cell Metab 29: 611–626.e5. 10.1016/j.cmet.2018.11.015 30581119

[bib56] Vergadi E, Ieronymaki E, Lyroni K, Vaporidi K, Tsatsanis C (2017) Akt signaling pathway in macrophage activation and M1/M2 polarization. J Immunol 198: 1006–1014. 10.4049/jimmunol.1601515 28115590

[bib57] Viola A, Munari F, Sánchez-Rodríguez R, Scolaro T, Castegna A (2019) The metabolic signature of macrophage responses. Front Immunol 10: 1462. 10.3389/fimmu.2019.01462 31333642 PMC6618143

[bib58] Wang F, Zhang S, Vuckovic I, Jeon R, Lerman A, Folmes CD, Dzeja PP, Herrmann J (2018) Glycolytic stimulation is not a requirement for M2 macrophage differentiation. Cell Metab 28: 463–475.e4. 10.1016/j.cmet.2018.08.012 30184486 PMC6449248

[bib59] Wang Q, Lu D, Fan L, Li Y, Liu Y, Yu H, Wang H, Liu J, Sun G (2019) COX-2 induces apoptosis-resistance in hepatocellular carcinoma cells via the HIF-1α/PKM2 pathway. Int J Mol Med 43: 475–488. 10.3892/ijmm.2018.3936 30365092

[bib61] Yu Q, Wang Y, Dong L, He Y, Liu R, Yang Q, Cao Y, Wang Y, Jia A, Bi Y, (2020) Regulations of glycolytic activities on macrophages functions in tumor and infectious inflammation. Front Cell Infect Microbiol 10: 287. 10.3389/fcimb.2020.00287 32596169 PMC7303283

[bib62] Yunna C, Mengru H, Lei W, Weidong C (2020) Macrophage M1/M2 polarization. Eur J Pharmacol 877: 173090. 10.1016/j.ejphar.2020.173090 32234529

